# Sociodemographic predictors of attendance at a Scottish pain management programme

**DOI:** 10.1177/2049463720970579

**Published:** 2020-11-06

**Authors:** FR Moore, L Williams, M Dunbar

**Affiliations:** 1 Phoenix Centre, Raigmore Hospital, Inverness, UK; 2 New Victoria Hospital, Glasgow, UK

**Keywords:** Pain management programme, socioeconomic deprivation, inverse care law, health care, programme attendance

## Abstract

We examined relationships between various sociodemographic factors and attendance at the Glasgow Pain Management Programme (n = 2899 from 2011 to 2019). We tested for associations between gender, age and socioeconomic deprivation of patients who were invited to attend, and uptake to a programme when invited, attendance at screening assessment, eligibility, adherence and attendance at 3- and 6-month reviews. Uptake was significantly higher for patients from more affluent areas (95% confidence interval (CI) = 0.93–0.99, p = 0.002) and for older patients (95% CI = 0.98–0.99, p = 0.006), although effect sizes were very small. Patients were significantly more likely to be assessed as suitable if they were younger (95% CI = 0.98–0.99, p = 0.013) or female (95% CI = 0.55–0.84, p < 0.001). Attendance at sessions and at 3- and 6-month reviews was higher for patients from more affluent areas (95% CI = 1–1.09, p = 0.001, and 95% CI = 1–1.1, p = 0.044 respectively). We argue that there are multiple potential explanations for these findings and that future work should attempt to determine whether these patterns replicate in other populations and to determine any modifiable causes.

## Introduction

A variety of sociodemographic factors have been shown to be associated with the risk of developing chronic pain. Greater age is associated with a higher prevalence of chronic pain.^1^ Men are less likely to report chronic pain and, even when adjusting for this lower incidence,^2^ are less likely to seek treatment for pain than are women.^1,2^ Being socioeconomically disadvantaged is also associated with an increased incidence of chronic pain.^3^ Among those with pain, those who are socioeconomically deprived report the pain to be more severe and disabling.^4^ Reported severity of persistent pain following caesarean section, for example, was greater in Scottish women from areas of greater deprivation.^5^ Similarly, Clement et al.^6^ found deprivation to be positively correlated with the number of pain comorbidities and reported severity in Scottish patients undergoing knee replacement surgery for symptomatic osteoarthritis.

These sociodemographic variables are associated with a number of other health-related factors, including access and response to treatment and, ultimately, to variations in mortality.^7^ The reasons for these differences are undoubtedly complex and may or may not be open to modification. One factor that is open to change is ensuring that access to healthcare is fair and equitable across different sociodemographic groups. However, it has long been recognised that an ‘inverse care law’,^8–10^ which states that good medical care is least available to the populations that most need it, sometimes operates. In line with this is evidence of poorer provision of health services in deprived areas. For example, patients in more deprived areas in the West of Scotland reported slower access to care, lower satisfaction with access and shorter clinical encounters.^11^ In addition, deprivation may influence the likelihood of attending healthcare services and appointments. For example, patients with diabetes in Tayside, Scotland, were less likely to attend retinal screening if they came from an area of high deprivation^12^ and uptake of influenza immunisation was inversely correlated with deprivation across the United Kingdom.^13,14^

Here we sought to identify key sociodemographic factors which influence uptake of invitation to attend, and attendance at, the Glasgow Pain Management Programme (GPMP). The catchment population for this programme includes some of Scotland’s most socially deprived areas. We collected data on the gender, age and socioeconomic deprivation of patients who were invited to attend the GPMP, and tested for associations between these with uptake of a programme when invited, attendance at screening assessment, eligibility, adherence and attendance at 3- and 6-month reviews.

## Methods

### Setting

Our catchment area was the Greater Glasgow and Clyde NHS-Scotland Health Board. The board is the largest in the United Kingdom and provides healthcare to 1.14 million people. Patients are referred to the programme by the secondary care multidisciplinary pain team across three Glasgow hospitals. Inclusion criteria (see Appendix 1) are in line with those suggested by the British Pain Society.^15^

### Programme

The GPMP is an outpatient rehabilitation programme for people living with chronic pain that is delivered along Acceptance and Commitment Therapy^16^ principles. It consists of either 10 or 12 weekly group sessions each lasting two and a half hours, delivered by a multidisciplinary team drawn from clinical psychology, nursing and physiotherapy. Patients attend review sessions with the team to evaluate pain management 3 and 6 months after the end of their programme.

### Measures

The age, gender and postcode of all patients who were invited to attend the GPMP were recorded.

Using the Scottish Index of Multiple Deprivation (2016; SIMD-16(2016,SIMD-16),^17^ patients’ postcodes were each assigned a rank. SIMD-16 is the Scottish Government’s official tool to identify areas of multiple deprivation in Scotland, using multiple indicators to rank postcode areas from most deprived (ranked 1) to least deprived (ranked 6976). SIMD ranks are constructed from 30 ‘indicators of deprivation’, which reflect a variety of social, health and geographical issues at the local level. Each local level, or data zone in SIMD parlance, contains roughly the same number of people. Therefore, measurement is not taken at the individual or even household level; rather it reflects the daily realities of the 760 people, on average, who live in each data zone. These 30 indicators are then grouped into seven domains, which are weighted and combined to produce a total score which is then given a rank across the 6976 data zones that comprise Scotland. Here, we used SIMD-16^17^ deciles as our unit of analysis in inferential statistical tests.

The following were recorded: (1) uptake of invitation to screening assessment (yes or no), (2) attendance at screening assessment (yes or no), (3) eligibility (yes or no), (4) adherence (percentage of GPMP sessions attended), (5) attendance at 3-month review (yes or no) and (6) attendance at 6-month review (yes or no).

### Statistical analysis

Multiple regression models were used to determine whether age, gender, or SIMD-16^17^ deciles independently predicted the six outcome measures described above (Table 1). Variables that were found to be statistically significant predictors of the above outcomes were followed up with planned post hoc tests (t-test and chi-square). Analyses were conducted using IBM SPSS, version 22.

**Table 1.** Percentage uptake of invitation to screening assessment, attendance at screening assessment, eligibility and attendance at 3- and 6-month reviews, and mean adherence (with standard deviations in parentheses) organised by gender, age group and SIMD quintile^1^.

	Gender		Age groups				SIMD quintiles				
	Male, n = 775	Female, n = 2115	18–42, n = 718	43–50, n = 718	51–57, n = 718	58–87, n = 718	1, n = 1271	2, n = 488	3, n = 406	4, n = 386	5, n = 348
Percentage uptake of invitation to screening assessment (of 2899 recipients)	68.1	68.5	66.6	63.0	71.5	72.8	65.9	69.5	67.0	69.2	76.7
Attendance at screening assessment (% of those 1982 patients who opted in for assessment)	98.1	97.9	97.1	97.9	98.2	98.4	97.7	97.1	98.2	98.5	98.9
Eligibility (% of the 1941 patients who attended screening assessment)	62.5	52.9	63.2	63.7	64.6	55.1	59.3	67.2	64.2	63.8	57.4
Adherence (% of weekly sessions attended by the 827 eligible patients who attended a programme; with standard deviations in parentheses)	86.67 (33.26)	85.71 (29.39)	86.13 (27.88)	82.69 (32.7)	88.24 (27.91)	86.64 (33.88)	81.93 (34.84)	91.25 (25.69)	85.56 (28.28)	81.88 (29.66)	92.56 (25.15)
Attendance at 3-month review (% of the 827 eligible patients who attended a programme)	43.7	49	47.2	50.8	52.8	41.6	44.13	51.7	42	44.7	60.3
Attendance at 6-month review (% of the 827 eligible patients who attended a programme)	30.4	34.5	33.1	36.9	33.8	32.7	32.9	35	27.2	28.2	44.9

1 SIMD-16^17^ quintiles are used here to describe proportions of patients in categories of socioeconomic deprivation (i.e. we considered SIMD-16 ranks or deciles to be too fine-grained to be meaningful here). An SIMD-16^17^ quintile of 1 represents greatest deprivation.

## Results

For all analyses, continuous variables (i.e. SIMD-16^17^ decile, age and adherence) generated skewness and kurtosis coefficients that were within parameters of normality for regression.

### Patients

All patients who were referred to the GPMP between 2011 and May 2019 were sent an invitation to attend an assessment appointment (n = 2899; female n = 2115 (73.2%); male n = 775 (26.8%); not reported n = 9; age: mean = 50, SD = 11.79). Non-responders were sent a further reminder. General Practitioners of non-responders were contacted individually, first to check that we had the correct patient address, and then to ask the General Practitioners to give patients a leaflet and further invitation to opt in to the GPMP.

### Socioeconomic profile

**Figure 1.**
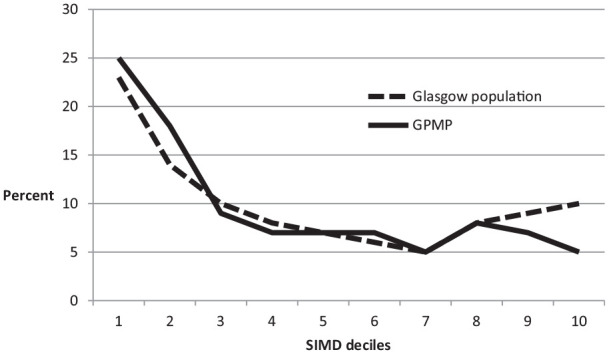
Distribution of SIMD-16^17^ deciles in Greater Glasgow and Clyde and the GPMP sample.

The distribution of SIMD-16^17^ in the sample matched that of the population of the catchment area of Greater Glasgow and Clyde, as shown in Figure 1. A chi-square test revealed that the SIMD-16^17^ ranks of our sample (n = 2899) did not differ significantly from those of Greater Glasgow and Clyde (n = 1.14 million; 17; chi-square = 4.75, df = 9, p = 0.86).

### Uptake of invitation to screening assessment

Of the 2899 patients who received an invitation, 1982 (68.4%) opted in to assessment for the programme and 917 (31.6%) did not. Of these, age was not available for 15 and gender was not available for nine patients. Independent samples t-tests revealed that SIMD-16^17^ deciles did not differ significantly between patients for whom age (t(1980) = –1.68, p = 0.093) or gender (t(1980) = –1.44, p = 0.15) were missing. Therefore, we included all patients in analyses.

**Figure 2.**
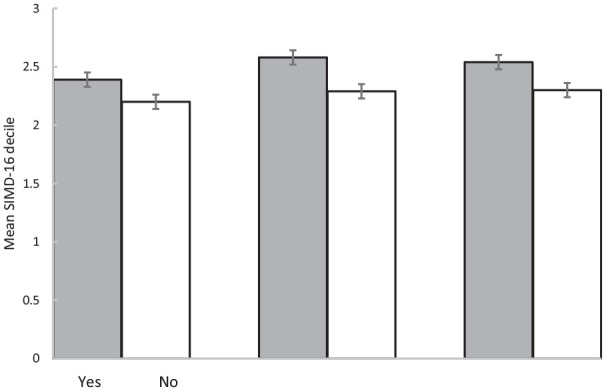
Mean SIMD-16^17^ decile of (a) those 2899 patients who received an invitation and who did and did not opt in to a screening assessment (i.e. uptake), (b) those 827 patients who attended a programme and did and did not attend a 3-month review and (c) those 827 patients who attended a programme and did and not attend a 6-month review (error bars are ±1 SE).

**Figure 3.**
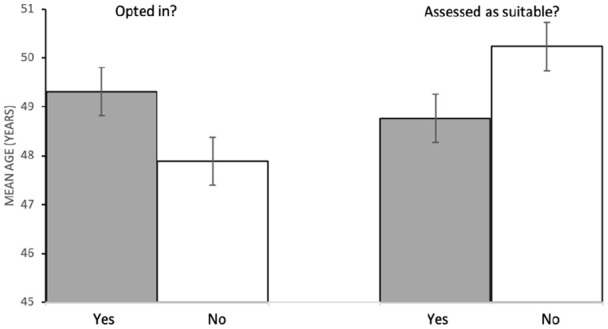
Mean age (years) of patients who (a) did and did not opt in for assessment to the programme and (b) were and were not assessed as suitable for the programme (error bars are ±1 SE).

In binary logistic regression with age and gender as covariates (Nagelkerke R^2^ = 0.01), there was a very small, significant, relationship between SIMD-16^17^ decile and uptake of invitation to screening assessment (Exp(B) = 0.96 (95% confidence interval (CI): 0.93–0.99), p = 0.002). Figure 2 shows that uptake was greater of patients from less-deprived (i.e. higher SIMD-16^17^ decile) regions. There was a very small, significant, relationship between age and uptake (Exp(B) = 0.99 (95% CI: 0.98–0.99), p = 0.006), such that those who responded were older than non-responders (see Figure 3). Gender (Exp(B) = 1.01, p = 0.889) did not significantly predict uptake.

### Attended screening assessment

Of the 1982 patients who opted in for assessment, 1941 patients (97.93%) attended for screening assessment and 41 (2.1%) did not. Binary logistic regression with age and gender as covariates (Nagelkerke R^2^ = 0.01) revealed no significant associations between gender (Exp (B) = 1.15, p = 0.712), age (Exp (B) = 1.01, p = 0.398) or SIMD-16^17^ decile (Exp (B) = 1.06, p = 0.297), and whether or not patients attended screening assessment.

### Eligibility

Of the 1941 patients who attended assessment, 1163 (59.9%) were classified by two members, from different professions, of the multidisciplinary team as suitable for the programme and offered a place (see Appendix 1 for details of assessment criteria) and 723 (37.2%) were not. Data on eligibility were missing for the remaining 55 (2.8%) of patients, so they were excluded from analysis.

Binary logistic regression with age and gender as covariates (Nagelkerke R^2^ = 0.02) revealed a very small, significant, relationship between eligibility and age (Exp (B) = 0.99 (95% CI: 0.98–0.99), p = 0.013) and a medium, significant, relationship with gender (Exp (B) = 0.68 (95% CI: 0.55–0.84), p < 0.001). Figure 3 shows that the group of patients who were assessed as eligible for the programme were younger than those who were assessed as ineligible. Post hoc chi-square analysis confirmed that more women were assessed as eligible than was expected (observed = 885, expected = 849.4) and more men were assessed as not eligible than were expected (observed = 228, expected = 192.1). That is, women were more likely than chance to be assessed as eligible, and men were less likely than chance to be assessed as eligible. SIMD-16^17^ decile did not significantly predict assessment of eligibility (Exp (B) = 0.99, p = 0.92).

### Attendance

Of the 1163 eligible patients, 827 (71.1%) attended at least one session of a programme and 336 (28.9%) did not. Binary logistic regression with age and gender as covariates (Nagelkerke R^2^ = 0.01) revealed no significant associations between gender (Exp (B) = 1.29, p = 0.113), age (Exp (B) = 0.99, p = 0.091) or SIMD-16^17^ decile (Exp (B) = 0.99, p = 0.752), and whether or not patients joined the programme.

Of the 827 patients who joined a programme, data for adherence were missing for 252 (30.5%) patients. These were excluded from the analysis. Percentages of sessions attended were calculated for the remaining patients. Multiple linear regression with age and gender as covariates (Adj R^2^ = 0.01, F(3, 569) = 2.47, p = 0.061) revealed no significant relationships between gender or age with adherence (both p > 0.1), but a small, significant, positive relationship between SIMD-16^17^ decile and adherence (B = 0.09, 95% CI: 0.11-2.19, p = 0.031). That is, patients from areas with relatively lower deprivation attended more sessions.

### Attendance at review

Of the 827 who joined, 381 (46.1%) attended a 3-month review and 448 (53.9%) did not attend. Binary logistic regression with age and gender as covariates (Nagelkerke R^2^ = 0.03) showed that gender and age did not predict attendance at 3-month review (p > 0.2). SIMD-16^17^ decile predicted attendance (Exp (B) = 1.09, p < 0.001). Figure 2 shows that the group who attended the 3-month review came from areas of lower deprivation than those who did not attend.

Two-hundred and eighty-five (34.5%) attended a 6-month review and 542 (65.5%) did not attend. Binary logistic regression with age and gender as covariates (Nagelkerke R^2^ = 0.01) showed that gender and age did not predict attendance at 6-month review (p > 0.06). SIMD-16^17^ decile predicted attendance (Exp (B) = 0.99, 95% CI: 1–1.1, p = 0.044). Figure 2 shows that the group who attended the 6-month review came from areas of lower deprivation than those who did not attend.

## Discussion

We tested for relationships between age, gender and socioeconomic deprivation, with attendance at a pain management programme. We found that, in terms of the distribution of patients across SIMD-16^17^ deciles, the sample was a close match to the distribution across Greater Glasgow and Clyde as a whole. Patients from areas of greater socioeconomic deprivation were less likely to accept an invitation to a screening assessment and, if they did, they attended fewer sessions and were less likely to attend 3- and 6-month review appointments, than those from more affluent areas. Attendance at review appointments was very low (<50%), which is consistent with well-established high-attrition rates at group interventions.^18,19^ Older patients were more likely to uptake an invitation to screening assessment, but less likely to be assessed as eligible, than were younger patients. Women were more likely than men to be assessed as eligible.

While the SIMD-16^17^ deprivation profile of patients referred to the GPMP closely matched that of the population of Glasgow as a whole, it is perhaps premature to suggest that those from more deprived backgrounds are not discriminated against in terms of access to the GPMP. This hesitancy is due to the fact that rates of chronic pain are higher among those from more socioeconomically deprived areas^3^ and, therefore, it would be predicted that the proportion of the GPMP sample that came from the most deprived postcodes should be higher than the population base rate, which we did not find.

There are a number of points on the journey between the onset of chronic pain and referral to the GPMP where factors could intervene to prevent referral. One possible explanation is that despite the higher prevalence of chronic pain, those in more deprived populations might be less likely to seek help from their general practitioner, although the limited evidence available does not suggest that this process is in operation among people with hip and knee pain.^20^ Then, when help is sought, gatekeepers, such as general practitioners and other primary care health workers, might be less likely to identify the problem among those from deprived areas, and when identified, they might be less likely to refer onto specialist pain services. There is some evidence that referral rates to secondary care for hip pain are lower among those from areas of higher deprivation^21^ so this might provide a partial explanation. This difference may well reflect a number of more general processes that have been observed in consultations in primary care, such as shorter consultation times and less ‘patient enablement’ among patients from poorer backgrounds when compared to their wealthier counterparts.^10^

Our results demonstrated a relationship between socioeconomic deprivation uptake of an invitation to attend a pain management programme, and of attendance at sessions and at follow-up. These findings contrast with the results reported in a recent systematic review that looked at factors associated with drop-out from interdisciplinary pain management programmes.^22^ This review found that the related variables of education and social status did not predict drop-out, regardless of whether these variables were examined in univariate or multivariate analyses. Why our own findings differ from those produced by this review requires explanation.

The most obvious candidate in explaining this lack of agreement is the difference in measurement of socioeconomic disadvantage employed by the studies in that review (namely, education and social status) and the SIMD-16^17^ measure used here. One Scottish data linkage study with a very large sample^23^ investigated the factors that predicted the failure to attend multiple General Practice appointments. The authors reported that SIMD-16^17^ rank was one of the more potent predictors, out of list that also included age, sex and various practice factors. Similarly, an analysis conducted by NHS Health Scotland,^22^ of who fails to attend their first appointment following referral to secondary care, found that being in the most deprived SIMD-16^17^ decile more than doubled the risk of not attending. To understand why SIMD-16^17^ might predict attendance, when education and social status apparently do not, it is helpful to better understand the SIMD-16^17^ measure.

Locality health statistics contribute 14% to the total SIMD-16^17^ score. These statistics include the standardised mortality ratio, the percentage of the population in each postcode that is currently being prescribed medication for anxiety, depression and psychosis, standardised figures representing emergency stays in hospital as well as other population statistics that reflect rates of drug and alcohol-related admissions to hospital. Thus, the SIMD-16^17^ reflects the extra burden of disease in patients from more socioeconomically deprived areas. It is not difficult to imagine ways in which this extra burden could interfere with the ability to attend healthcare appointments. Indeed, another large Scottish data linkage study^23^ found that the number of long-term conditions a patient has was also a strong predictor of the risk of failing to attend general practice appointments. Geographical issues, such as the time taken to travel to local resources, including local general practitioner practices, also contribute to the total SIMD-16^17^ score and could reflect the difficulties in getting to appointments more generally.

The SIMD-16^17^ also reflects financial constraints, in that the levels of unemployment and the percentage of the locality that are in receipt of state benefits, both contribute to the weighted total score. It is not difficult to imagine that the financial costs of attendance at outpatient appointments are more relevant to those whose income is most limited. Similarly, there may be greater stress associated with juggling the demands of work and attendance at sessions, particularly for those with unstable or ‘zero hours’ employment. Regardless of the reasons for non-attendance in our sample, the results reported here add to the evidence suggesting that one way in which deprivation contributes to ill health is the difficulties it creates in accessing healthcare.

Increasing age was associated with a greater uptake, but a reduced chance of being assessed as suitable. It is well documented that older age is associated with an increased likelihood of appointment attendance^24–26^ although the data reported here failed to find any association between GPMP attendance and age. However, the increased uptake in older patients might reflect similar processes. The fact that older patients were less likely to be assessed as suitable for a place on the programme may reflect processes related to age discrimination. Age discrimination in health care has been well documented and attitudes among some healthcare staff have been shown to be discriminatory.^27^ We are not suggesting that this is what has taken place here, although such attitudes can be unconscious. There are also a number of other possible reasons for this difference, not least of which is the greater burden of ill health and disability among older people. A significant component of this extra burden is the difficulties that are brought about by the treatment that these conditions require. These treatment burdens include multiple healthcare appointments, the use of multiple medications and the need to self-care.^28^ These burdens have been shown to affect treatment adherence, including attendance at medical appointments.^28,29^ It is also worth noting that the outcome from assessment is not simply a binary, yes or no decision. Instead, decisions are made as to the most appropriate service for the patient’s particular needs and circumstances, which often involves referral onto other services.

To summarise, we found relationships between socioeconomic deprivation and uptake of a pain management programme, and of attendance at sessions. We argue that there are multiple potential explanations for this, and that future work could try to examine the independent contributions of income, geographical constraints and the burden of associated ill health. We would also encourage work looking at interventions that might address this unequal access. Encouragingly, we did not find evidence to support an effect of patient socioeconomic deprivation on clinician assessment of suitability for the programme. This suggests that lower attendance rates among patients from areas of high deprivation are not due to discrimination in the availability of the service, but rather due to factors which are more likely to prevent attendance from those experiencing greater deprivation.

## Supplemental Material

Appendix – Supplemental material for Sociodemographic predictors of attendance at a Scottish pain management programme Click here for additional data file. Supplemental material, Appendix for Sociodemographic predictors of attendance at a Scottish pain management programme by FR Moore, L Williams and M Dunbar in British Journal of Pain
