# Supplementary material for: Sociodemographic predictors of attendance at a Scottish pain management programme
Source: Br J Pain. 2020 Nov 6;15(4):393–400. doi: 10.1177/2049463720970579 (PMC8611294; doi:10.1177/2049463720970579)
Supplement: Appendix – Supplemental material for Sociodemographic predictors of attendance at a Scottish pain management programme [file Appendix.docx]

Appendix 1. Assessment criteria for the Glasgow Pain Management Programme.

Inclusion:

Non-malignant chronic pain of over three months duration that causes distress, disability or a reduced quality of life.

Appropriate medical screening to exclude treatable disease has been conducted prior to referral.

Willingness to participate in a group intervention.

Exclusion:

Severe disability such that the basic requirements of attending exceed the participant’s current capacity (consider onward referral to specialist residential PMP settings or for individual physical and/or occupational therapy).

Extreme fatigue or impaired concentration or cognition, such that these undermine the potential for treatment gains (consider onward referral to appropriate services).

Psychological or psychiatric problems which require urgent attention, or are of such severity that they are likely to undermine attendance and/or treatment gains.

Other considerations:

Self-professed (i.e. not based solely on clinician’s judgements) difficulties with motivation to engage in cognitive or behavioural strategies. Following careful discussion with participant, consider onward referral to other educational or low intensity interventions with the aim of improving motivation and skills for behaviour change and encourage re-referral once these interventions are complete.
